# Understanding decision processes in becoming a fee-for-hire service provider: A case study on direct seeded rice in Bihar, India

**DOI:** 10.1016/j.jrurstud.2021.09.025

**Published:** 2021-10

**Authors:** Brendan Brown, Arindam Samaddar, Kamaljeet Singh, Ava Leipzig, Anurag Kumar, Pankaj Kumar, Deepak Kumar Singh, Ram Malik, Peter Craufurd, Virender Kumar, Andrew McDonald

**Affiliations:** a International Maize and Wheat Improvement Center, Kathmandu, Nepal; b International Rice Research Institute, New Delhi, India; c International Maize and Wheat Improvement Center, Patna, India; d International Rice Research Institute, Los Baños, Philippines; e School of Integrative Plant Science Soil and Crop Sciences Section, College of Agricultural and Life Science, Cornell University, USA

**Keywords:** Directly seeded rice, Zero tillage, Service provision, Sustainable intensification, Conservation agriculture, Qualitative methods, Decision-making dartboard

## Abstract

While Direct Seeded Rice (DSR) has numerous potential benefits to smallholder farmers in the Eastern Gangetic Plains of South Asia, it's out-scaling has been limited by both a lack of demand by farmers and limited supply of DSR services by machinery owners. This contrasts with the comparatively more rapid scaling of zero tillage wheat in the region. This trend is yet to be fully explored, particularly when focus has been placed almost exclusively on understanding DSR adoption though the lens of farm-level agronomic, economic and environmental performance. Given that limited DSR service provision is likely to be governed outside of these considerations, this study explores with zero tillage drill owners the decision processes they apply in deciding how to use their zero tillage drills. Respondents highlight a complex web of interrelated considerations that highlight the additional complexities of DSR as compared to existing practices. Using a novel ‘Decision-making Dartboard’ qualitative framework, these complexities are unpacked and a set of potential changes to the assumed theory of change for DSR scaling are identified, including considerations for selection of potential DSR service providers and responsibilities for promotion and extension of DSR to overcome the prevalent negative perceptions of DSR held broadly across the communities explored. The proposed framework and analysis process are also potentially useful for exploration of other farmer decision making processes more broadly.

## Highlights

• Limited service provision is a key constraint to scaling Direct Seeded Rice.
• No work explores why Zero Tillage Drill owners don't use it for Rice.
• Issues exist with both technology and generically providing services.
• Collectivism or service packaging may resolve some of these issues.
• Novel framework provides unique qualitative analysis on decision making.

## Graphical abstract

**Figure:**
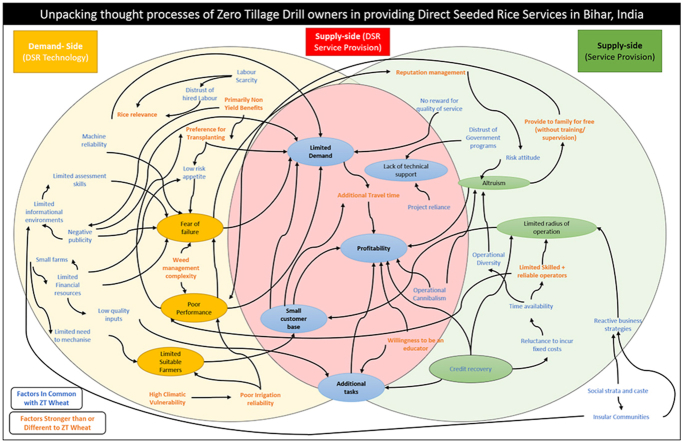

## 1 Introduction

Population growth coinciding with the Green Revolution increased labour supply and made labour-intensive rice systems possible (Pandey and Velasco, 2002). This change in labour supply, coupled with improved transplanting methods and frequent irrigation led to a shift in cultivation methods from broadcasting to Puddled Transplanted Rice (PTR) systems (Pandey and Velasco, 2002; Kaur and Singh, 2017). PTR encompasses growing rice seedlings in a nursery, regularly irrigating fields, and transplanting seedlings into puddled fields. PTR currently dominates rice cultivation methods in both the Indo-Gangetic Plains and globally (Pandey and Velasco, 2002; Kakumanu et al., 2019).

While initially catalysing vast improvements in rice yields, more recently the productivity of rice has stagnated across the Indo-Gangetic Plains, and even declined in certain areas (Saharawat et al., 2010). This is particularly concerning given rice provides 43% of the calorie requirement for over two-thirds of the Indian population (Kaur and Singh, 2017; Singh et al., 2019), and the demand for rice in India is expected to rise 26% by 2035 to meet population growth demands (Kumar and Ladha, 2011). PTR was initially enabled by abundant labour, which is now increasingly scarce and expensive in the Indo-Gangetic Plains where labour dynamics are often driven by opportunities in non-agricultural sectors (Kakumanu et al., 2019). This is particularly problematic as PTR requires intensive labour in concentrated periods of time, specifically during the transplanting stage, resulting in labour bottlenecks (Malik et al., 2015; Kaur and Singh, 2017).

While such labour constraints may be partly overcome through mechanised PTR (Guru et al., 2018), it is unlikely to address the climatic concerns of this cultivation practice, particularly due to the prevalence of PTR in combination with puddling. Due to the anaerobic soil conditions, methanogenic activity occurs (Kaur and Singh, 2017; Kakumanu et al., 2019), making PTR a notable source of methane emission – a potent greenhouse gas that contributes 10–20% of global annual methane emissions (Kakumanu et al., 2019). India alone releases 4.5 million tons of methane annually through rice production (Maharjan et al., 2012). PTR has also been shown under certain conditions to adversely affect soil productivity through compaction and salinity build-up and can lead to subsequent wheat yield loss in PTR fields of 8% (Malik et al., 2015), as well as delay wheat planting which can cause as significant as 1% yield loss per day of delay beyond optimal planting window of November 15 (Kaur and Singh, 2017; Mishra et al., 2017).

To address the need for alternatives to PTR, a growing body of research has explored the potential of Directly Seeded Rice (DSR), which more recently has been promoted using a Zero-Tillage (ZT) drill to plant rice seed in non-puddled fields. DSR has been shown to address many of the above limitations of PTR. DSR avoids nursery raising, seedling uprooting, transplanting, and puddling thereby leading to labour reductions of up to 50% (Kumar and Ladha, 2011) while increasing planting date flexibility which reduces bottlenecks in labour supply (Kaur and Singh, 2017; Devkota et al., 2020). DSR also reduces fuel requirements by 34–60% compared to PTR (Kumar et al., 2015). As DSR can be implemented without puddling, irrigation water use can also be reduced by between 12% and 33% (Kumar and Ladha, 2011). This avoidance of puddling also enables a reduction in methane emissions by up to 85% (Kumar and Ladha, 2011).

When DSR is implemented in appropriate agro-ecologies (i.e. lowlands with assured irrigation and levelled land) where weed pressure is low, the mentioned benefits are achieved with mostly stable yields (Pandey and Velasco, 2002; Mishra et al., 2017; Devkota et al., 2020). While some studies have identified yield reductions in DSR systems, Farooq et al. (2011) and Xu et al. (2019) argue that well-managed fields usually overcome such productivity limiting issues. Even if yields are reduced, the economic benefits usually outweigh this though higher economic returns (Kakumanu et al., 2019; Devkota et al., 2020), with DSR cost savings of up to USD 149/ha (Singh et al., 2020). Thus, DSR is generally seen as a worthwhile economic investment, even accounting for potential yield reductions.

Enabling smallholder mechanisation, and particularly DSR uptake, requires a functional fee-for-service provision sector, as most smallholder farmers will never achieve the required financial and knowledge requirements to individually own and operate a four-wheel tractor driven ZT drill. Service provision has been crucial to expanding adoption of other agricultural machinery (Targeting, 2014), given that Keil, D'Souza and McDonald (2016) found that only 8% of non-service provider households owned a four-wheel tractor and Erenstein and Farooq (2009) found that 60% of ZT wheat adopters rely on ZT drill service providers. If the assertions made by Keil et al. (2016) that ZT wheat was primarily governed by [1] proximity to service providers; and [2] awareness, hold true for DSR, then ensuring drills are made available by DSR service providers is likely a key driver to enabling more widespread adoption of DSR by farmers across the region.

Wheat planted with the ZT drill now covers more than 3 million hectare in the Indo Gangetic Plains and continues to expand (Kumar and Ladha, 2011). DSR requires the same ZT drill used to plant ZT wheat, and the common theory of change is predicated on ZT drill owners utilising their ZT drill for Monsoon rice (i.e. for DSR). Such service providers who provide ZT wheat services have already invested in the requisite machinery and have established customer networks, enabling them to effectively add an additional season of potential profit annually, yet this has not occurred. Project evidence from the Cereal Systems Initiatives for South Asia (CSISA) project suggests that while more than 5000 farmers are now using DSR for the establishment of rice across the Eastern Gangetic Plains through service providers, only 10% of ZT drill owning service providers are engaged in providing DSR services (personal communication, CSISA Donor Report 2017). This means that the potential benefits of DSR are not being widely experienced, and the decision making of ZT Drill owners warrants further investigation to ensure a more complete understanding as to how to stimulate the DSR service provision economy.

While analysis of the DSR service provision economy is sparse, some studies have analysed the ZT wheat service provision economy. Keil and Mitra (2017) found 85% of service providers interviewed three years apart continued to provide ZT wheat services, and 42% of interviewed service providers sought to expand their businesses through marketing, purchasing more drills, and increasing their area of operation. Keil, D'Souza and McDonald (2016) found that ZT wheat service providers in Bihar serviced an average of 20 clients annually and 20 ha of land. Their net profit ranged from INR 516/ha (with hired labour to run machines) to INR 593/ha (without hired labour). While this highlights the benefits of ZT wheat service provision, as yet there is no literature that explores the DSR service provision economy. This is particularly problematic given the comparatively rapidly expanding ZT wheat service provision economy, yet the stagnating DSR service provision economy.

While the literature had proven the theoretical benefits of DSR agronomically, economically and environmentally, there are few insights into the business decision making related to DSR service provision and, from a development perspective, what forms of support can catalyse the emergence of an inclusive DSR service economy for smallholder farmers. In particular, there is a need to understand the decision-making processes of ZT drill owners in their evaluation of DSR as a technology, as well as the context for providing (DSR) services in their communities. While the literature has explored characteristics of DSR adopters, identifying them as predominantly male (Mishra et al., 2017) under forty years old (Bhardwaj and Sidana, 2017), and likely to have larger operational areas (Singh et al., 2010), there is no exploration of ZT drill owner's decision making related to DSR. This is likely to be a complex web of reasons that is best explored though qualitative analysis of the decision-making processes of these ZT drill owners. Such analyses are absent from the literature, and hence the objective of this study.

This first of its kind study aims understand ZT drill owner's decision making to identify leverage points through which the DSR service provision sector can be catalysed, such that the benefits of DSR can be experienced by smallholder farmers across the Eastern Indo Gangetic Plains. This is achieved though semi-structured interviews on the contexts around decision making for both DSR and agricultural service provision. Our research question relates to what can be done to accelerate the progression of ZT Drill owners to: (a) implement DSR on their own fields; and (b) provide DSR services to others in their community. This research question is explored through the lived experiences and perspectives of local farmers and service providers in two districts in Bihar, India. Using a novel qualitative framework and semi-structured interviews with 43 ZT drill owners, the decision processes and considerations that lead drill owners to consider and implement DSR and provide DSR services are explored.

## 2 Methods

### 2.1 Location selection

This study was implemented in two districts in Bihar, India which have had ongoing scaling and extension programming for ZT wheat since 2009–10. Samastipur is approximately 80 km northeast of Bihar's provincial capital, Patna, while Buxar is approximately 125 km West of Patna. Both have substantial investment from developmental and governmental programs related to ZT wheat and DSR. These locations were originally targeted as they have substantial lowland areas suitable for the implementation of DSR (i.e. with less complex weed flora and comparatively more available irrigation facilities that enable earlier planting to avoid stand mortality). The two locations were selected based on the presence of a variety of ZT drill usage typologies (see section 2.2).

### 2.2 Participant selection

**Fig. 1:**
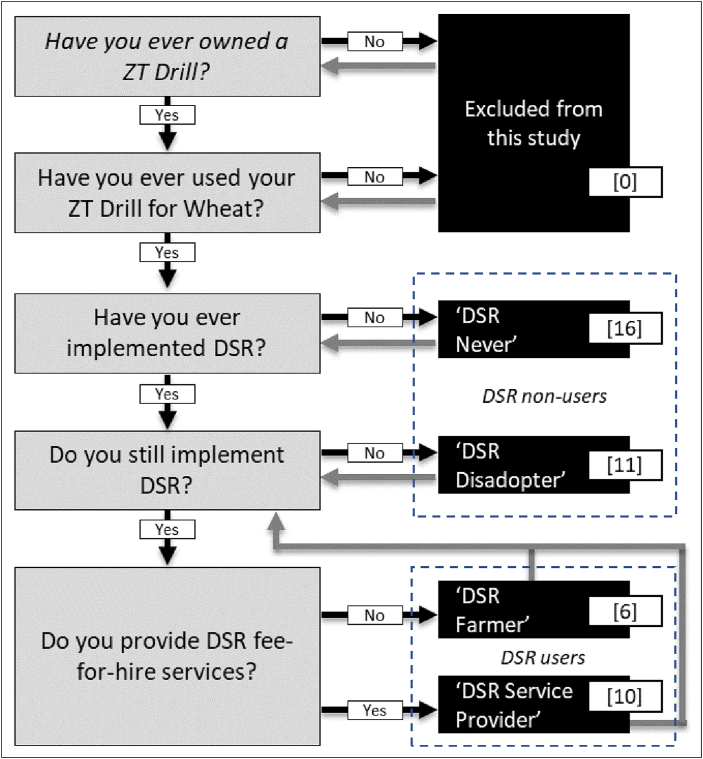
Selection criteria for inclusion in this study and typology classification. Brackets indicate number of respondents for each typology. Grey arrows indicate feedback loops that highlight future pathways, meaning no specified endpoint exists.

Answering our research question requires discussion with more than one type of ZT drill user. To ensure a diversity of experiences and outcomes with DSR, a set of typologies was developed based on a combination of the Stepwise Process of Mechanisation framework (Brown et al., 2021) and the Process of Agricultural Utilisation Framework (PAUF; Brown, Nuberg and Llewellyn, 2017b), to follow a logical stepwise process from never having used their ZT drill for DSR, to personal use, service provision and disadoption of DSR (Fig. 1). For inclusion in the study, a respondent needed to have personally purchased a ZT drill and have previously used it, either personally or as a service provider, for wheat in the *Rabi* (Winter) season. Within this, we purposively targeted four different DSR typologies (Fig. 1). In total, 43 respondents participated in this study.

### 2.3 Theoretical framework

**Fig. 2:**
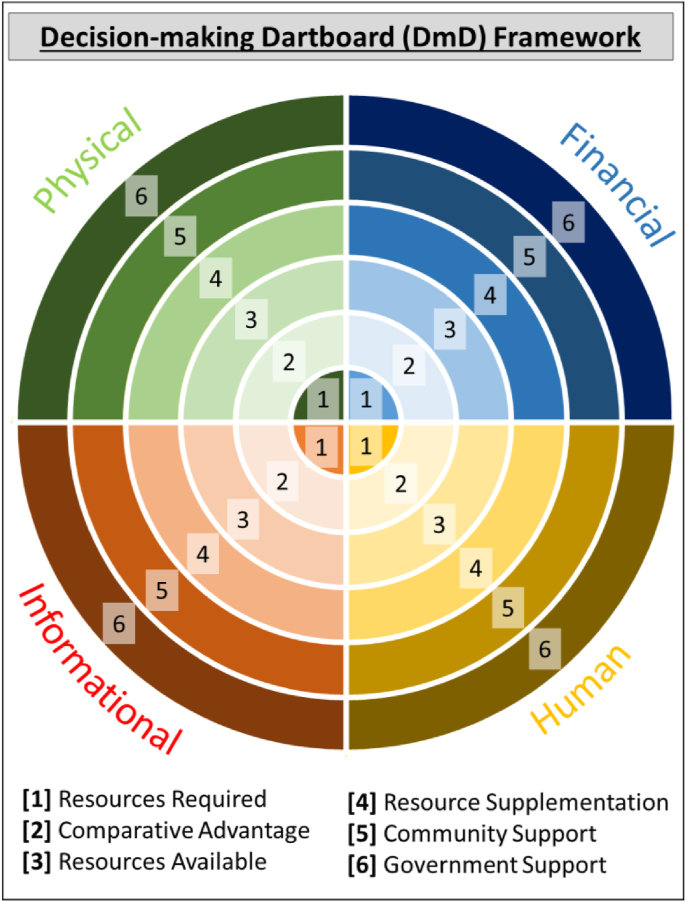
*The Decision-making Dartboard (DmD) Framework, an adaptation from the 3-dimensional Livelihood Platforms Approach framework* (Brown, Nuberg and Llewellyn, 2017a) *to 2 dimensions for increased visual clarity and ease of analysis.* (For interpretation of the references to colour in this figure legend, the reader is referred to the Web version of this article.)

In order to cover a wide spectrum of issues involved in decision making, this study applies a structured qualitative framework adapted from the Livelihood Platform Approach (LPA; Brown, Nuberg and Llewellyn, 2017a). The LPA has previously been applied to understand the decision-making processes of smallholder farmers in relation to conservation agriculture in Africa. The LPA builds on the sustainable livelihood framework to explore the uptake of agricultural technologies at individual, household, community, and institutional ‘platform’ levels (Anibaldi et al., 2021). Modifications here enable a deeper understanding of perceptions, abilities and enabling environments in which farmers make technological evaluations and decisions. This approach, termed the Decision-making Dartboard (DmD; Fig. 2) framework builds on existing LPA and sustainable livelihoods framework theory, but is adapted for deeper exploration of new contexts. The DmD, like the LPA, disaggregates key decision processes into six core questions across four asset categories, which when combined are used to explore the various considerations that individuals apply to reach their current typology outcomes.

### 2.4 Questionnaire development

The DmD framework was used to develop a semi-structured question schedule, which consisted of five modules. Module 1 contained a pre-screen to ensure compliance with selection criteria and attribution of typology, as well as basic demographic data (identification, location, education, farm and service provision information). Module 2 related to generic technological evaluation processes (risk preference and informational networks), learning processes, and extent of knowledge related to ZT Wheat and DSR. Module 3 related to the evaluation of DSR (comparisons of DSR to conventional practice and relevance to livelihood activities, as well as possible support to practice DSR). Module 4 explored the practical implementation of DSR (especially physical and human resource requirements) while module 5 explored the major constraints in performance and outcomes for DSR. Modules 2 through 5 were digitally recorded for later transcription, while KoboCollect ODK was used to collate information from module 1.

### 2.5 Survey implementation

Respondents were identified through a snowball sampling strategy, whereby locally based informants directed interviewers to DSR users, and those DSR users identified others in the community that fit with requested typologies. Efforts were made to not over sample any of the four typology types with the intention to characterize diversity rather than representativeness within communities.

The survey was implemented by a singular enumerator who was given an in-depth multi week training on qualitative data collection. Piloting and adaptation occurred before the enumerator began full implementation. The survey was implemented in Hindi, digitally recoded, and later transcribed to English for thematic coding. In total, 43 interviews were completed, with an average length of 39 and a half minutes and totalling 28.5 h of recorded Interview.

### 2.6 Analysis process

Pre-screening information was summarised using Microsoft excel, while all cleaned English transcripts were analysed in Dedoose qualitative software (Dedoose.com), and thematically coded using the DmD framework. The themes used for coding consisted of the 24 codes related to the DmD (6 levels by 4 resource types), with an additional 20 child-themes related to commonly raised topics (for example, communal human resources and issues of gender, social structure and caste, communal informational resources, business strategies, and weed management). In total, 3512 excerpts were coded into the above 44 themes. These themes were then analysed in line with the DmD to produce the given results.

### 2.7 Demographics of respondents

All respondents were male and had completed secondary education, with an average age of 46 years. All but three had at least five years of awareness of the ZT drill, and the majority first heard of ZT between 2009 and 2012. The majority of respondents bought their drills between 2012 and 2016. While 31 had taken ZT wheat training, only 4 had taken DSR training and six had taken no ZT training. 33 have provided ZT wheat services, and on average serviced 24 households on 51 acres annually. On the most recent season's data, respondents who provided DSR services were servicing an average of 18 farmers on 46 acres annually for with their ZT drill.

## 3 Results

### 3.1 Do comparisons with transplanted rice lead to limited DSR use and service provision?

#### 3.1.1 User perception of the multiple benefits of DSR

For DSR users, there was a common perception of stable (but importantly not improved) yields, yet a wide set of benefits that accrue from implementing a DSR system (e.g. “*Even if the yield is 80% or 90%, I will still prefer to plant it [DSR] … it doesn't matter to us because many of our problems and difficulties will be solved*” [C16]). The diversity of benefits raised are listed in Table 1 and reinforce the primary comparative benefits of DSR are not related to yield.

**Table 1:** Summary of non-yield benefits of DSR identified by DSR users.

Benefit	Representative quotation	ID
Lower production costs	“The yield is almost the same in both the systems, but as cost are less in DSR it is more beneficial in terms of income”	A1
Reduced tractor passage (and cost) even if tillage remains prior to use of ZT Drill	“The benefit of using ZT [drill for rice] is that it has reduced our tilling cost. In transplanting, we till our farm around five times using a cultivator but now we just do it two times with rotavator and then do the sowing with zero drill machine”	B8
Reduced water use	“Those who till their land [for puddling] require more water for irrigation and thus it is more costly”	A2
Precision fertiliser placement	“In traditional methods [PTR], fertilizers are spread across the field. But with the ZT drill, the fertilizers are dropped with the seeds. Thus, fertilizer is saved and weeds don't use the fertilizer”	B3
Reduced labour use	“The costliest thing to a farmer is labour and we save that with DSR”	C17
Reduced labour dependency	“DSR can saves a lot of labour cost and also all that hassle to arrange for labour and convincing them to come and work in our farm”	C7
Enables harvester use	“We are not able to use harvester in some of our lowland farm but with ZT drill now we can use a harvester. It won't become stuck or jammed”	C1
Early maturity and marketing benefits	“The crop is ready slightly earlier and since the crop harvest can take place sooner, there is no oversupply in the market and it will sell quickly. … I will get a good rate for it.”	C5
Complementary wheat planting	“When the [DSR] paddy is ready early, my plot will be empty due to which I will be able to plant wheat on time. If I plant wheat on time, my wheat will give higher yield. This leads to higher profit for me”	C9
Residual moisture for wheat crop	“During harvest if there is water the soil soaks it and then we can do the harvest. So when our rice field is cut, there is enough moisture in the soil that you can plant wheat straight away “	C1

#### 3.1.2 Non-user reasons for negative perceptions of DSR

While DSR non-users did not disagree on many of the benefits perceived by DSR users including key labour-saving benefits, they saw yield reductions as more sizable (e.g. “*Transplanting is more expensive. We sow seeds in seedbed, pay laborers, do puddling, sow seeds that need water, then apply DAP and then there are the expenses to transplant crop. All these things add up, but the establishment is good and crop yield is higher in comparison to DSR”* [C17]). In most cases, lower yield related back to issues with seed drop (e.g. “*In hand transplanting, one doesn't leave any spots empty, but in DSR, a lot spots are vacant where seeds fail to drop"* [A8]).

DSR non-users also tended to have negative limited or second-hand experiences of DSR weed management (e.g., “*There was so much of weed in his [DSR] farm that he did not get good germination and then did not get good yield*” [A4]), with often near total crop failure perceived to have occurred due to weed pressures. Disadopters in particular focused on weed management as a key determinant of disadoption (e.g., *“If we find a solution to the growth of weed, it would prove to be very beneficial to the farmers. We would stop practicing transplanting rice method as it proves to be extremely expensive to the farmers*” [C10]). The complexity of new management practices was also identified as of major concern. Puddling is known as a tried and tested method for weed control (e.g., “B*ecause we do puddling, it destroys all the weeds and there is very little weed growth after that*” [A3]), and was perceived as less complex compared to the multiple, sometimes new, activities required for DSR weed management (e.g. “*we use the herbicide and this kills everything except rice in DSR. But there has to be water/flood in the soil for it to work in PTR*” [A1]; “*to get the weeds under control by herbicide, we need sunshine for 48–60 hours. If it rains a little, only 50 percent of the weeds will die”*[C16]).

Beyond increased complexity, there were also concerns about the effectiveness of herbicides – both for information uncertainty (e.g. “*When we spray herbicide on the weeds, it proves to be ineffective … We cannot tell if there is deficiency in the medicine or deficiency in the doctor*” [C4]) and effectiveness (e.g. “*there are certain types of weed which do not get killed even when herbicides are sprayed*” [C13]). Complexities in weed management led to adaptation of DSR with the addition of tillage, complicating the known benefits of DSR such as reduced production costs and soil health benefits (e.g. “*The less we till the field, the better the seeds are sown, but we till the field a little bit just to stop the weeds*” [C10]). In turn, reduced DSR production costs were also often less recognized due to the persistence of tillage before DSR use (e.g. “*For DSR, we do two round of tilling and then do the sowing …. There isn't much difference in initial cost between the two*.” [C10]).

#### 3.1.3 DSR service provision

Overall, there were mixed perceptions about the comparative benefit of DSR service provision, especially when compared to the profitability of traditional tillage services. While there may be benefits to tractor life and diesel use, the profit margins were often perceived as less due to less field passes and hence DSR service provision was perceived as less profitable (e.g*. “DSR is profitable* *for farmers, but for service providers the traditional farming [PTR] is profitable. This is because every time he tills the fields a service provider's income increases …. In traditional methods, you need to till your fields at least 2–3 times before sowing any seed. So, from this multiple tilling, you will earn more money. But, in ZT and DSR, you can directly sow seeds, thus you can only collect one time”* [A5]). This often led to a continuation of tillage operations and then use of the ZT Drill for planting.

### 3.2 Do household resources and mindset lead to limited DSR use and service provision?

#### 3.2.1 Irrigation infrastructure

The requirement for irrigation infrastructure was consistently mentioned, because DSR requires early planting which increases vulnerability to variable rainfall which cannot be overcome without reliable early season irrigation (e.g., “*It [ZT] is good for wheat and also good for rice, but only if weather is favourable*” [B10]). Nearly all respondents agreed that if assured irrigation was available, DSR would be more widely adopted in their communities (e.g., “*If there are good irrigation facilities, then you will not have to tell any farmer to do DSR. Farmers will automatically start adopting*” [C9]).

#### 3.2.2 Land attributes

Specific types of land were also commonly identified across respondents, particularly when identifying why ZT wheat has increased in community usage while DSR has not. Participants particularly emphasised that level land was of increased importance for DSR as compared to ZT wheat (e.g. “*Because of flood water and the sediments it brings, our field are not levelled*” [B8]) and likewise in relation to water holding capacity (e.g. “*This land doesn't retain water. It rained yesterday but can you see any trace of water in the field?*” [C18]).

#### 3.2.3 Household tolerance for risk in the context of DSR

Despite respondents in this study being a subset that would be expected to be risk takers (as they have invested in ZT drills), there did appear some level of reluctance in accepting the perceived risk involved in DSR for users compared to non-users (Table 2). DSR users tended to perceive themselves as more willing to take risks and experiment, which may indicate a differential learning attitude to DSR non-users.

**Table 2:** Comparative examples of risk mindeset between DSR users (left) and DSR non users (right).

DSR user mindset quotations		DSR non-user mindset quotations	
“Sometimes one has to use his own mind and try new things. In the process we might incur a loss … it's my own farm, so I don't care if it's a failure but if it turns out great then I will show you”	A1	“I don't want to experiment. My mantra is that what I know I should do that and not take suggestions from anyone. If anybody gives me a suggestion, then I do listen to them but until my heart is satisfied. It is because if I make a mistake, I have to live with that for a year”	C22
“I am somebody who likes to do something new every year even though I may incur a loss in the process”	C1	“I can try DSR any day since I have a ZT machine. But a farmer has to think three times before taking one step as I don't want to lose my money”	B6
“It wasn't as good as I had expected, but I will try again. I am not one of those farmers who will give up easily. I might be a small farmer but I like to experiment and try out new things”	C13	“I had heard about the features of this machine but there is a fear of not being successful”	C21

#### 3.2.4 DSR service provision

Most service providers indicated a strong sense of self-reliance, especially for machinery maintenance (e.g. “A *farmer is half driver and half mechanic … Whatever machine a farmer owns or runs, he has 80% knowledge about its technical problem and how to fix it*” [C16]) and operation (e.g. “*For tilling I have a driver but when I use ZT machine, then I myself operate it as the driver is unable to properly do sowing with it.*” [A10]). Yet this use of personal time was a crucial divide between ‘farmer-only’ and ‘service provider’ respondents. Farmer respondents tended to identify a lack of time to provide services (e.g. “*I don't provide services. I have given it a thought, but I do not have time to rent out my services. I am extremely busy*” [C9]). This lack of time often led to farmers providing their ZT drill to others (particularly family members), though they did not operate the machine themselves. This also potentially relates to some of the technical challenges prevalent in the community based on limited trained operators and machinery performance issues. This was related to a complex web of issues encompassing identity, original intention, and altruism (Table 3).

**Table 3:** Traits evident in Service providers that were often absent in farmer only typologies.

Trait	Quotation from Service provider respondents	ID	Quotation from ‘Farmer-only’ respondents	ID
Prioritization of Farm Vs. Business	“I earn more from service provision. I am more of a service provider and give less attention to my own farm”	A3	“One can only think of providing services once he is free from his own work. I have my own very big farm that keeps me occupied. If I start providing services, then I cannot take care of my own farm.”	C22
Original intention	“At that time, it was a new thing. If I start doing it and get success, other farmers will notice and get interested and I can earn an income from it”	B9	“I have bought these machines for my own use. I don't have time to go on other people farm and provide services to them”	C15
Altruism	“There are many farmers who do not have money to pay for my services, I work free of cost for them. It is not important that every customer pays me for my services … I do it out of humanity as those farmers do not have money”	C11	“My primary motive behind buying this machine is to use it for our own farming.”	B8
Alternative livelihoods	“In our own agriculture, there's always fear of profit and loss. But, in services, there's only profit”.	B2	“If I was into one thing like DSR, I could have made an effort, but I have other businesses to look after as well”	C4
Future optimism	“The direction in which agriculture is moving is that at minimum cost and labour, we have to do farming which is machine based. I also want to walk in that direction”	A2	“The income is not increasing. I don't see any hope for the coming generation in agriculture”	C6

### 3.3 Does interaction with the community and communal resources lead to limited DSR use and service provision?

#### 3.3.1 Constraints hiring supplementary labour

Issues with hiring labour was the most discussed issue which has strong potential to drive adoption of DSR (e.g., “*30 years ago, after testing rice with seed drill, I left it. Then around 10 years ago there became a labour problem, so I returned*” [C1]). While scarcity of labour and associated cost increases were commonly raised as issues, there were various other issues primarily associated with a distrust in hired labour that were discussed by respondents (Table 4).

**Table 4:** Various issues associated with hired labour as discussed by respondents.

Issue	Representative quotation	ID
Increasing cost	“What happens is that during sowing time, labour demand exceeds labour supply so some farmers start offering more money to laborers to attract them. This season, people have paid as high as 150 rupee per ropani [usual price 100]”	B6
Reliability	“labour is a major issue in this area. They demand for higher prices and do not turn up for work for 5–6 days. They keep delaying starting work”	C16
Trust and Theft	“laborers are stealing from us. In an acre, around 400,000 seedlings should be planted but they plant about only 250,000. In a way, they are stealing maybe 100,000 in an acre. With ZT machine, we can plant around 700,000 seedlings in an acre. If around 200,000 of seedlings die before germination, even add 100,000 more to it, even then we still are left with 400,000 seedlings which are more than transplanting rice”	B2
Motivation and supervision	“Sometimes what happens is that if you yourself are not present at the farm, then the people you have hired don't do their work nicely.”	B7
Accuracy of implementation	“A crop is to be sown at a distance of 5 cm, but laborers would plant them unevenly, from 8 cm to 10 cm. A machine would sow the seeds according to a standard distance, around 5–6 cm. As a result, we will get plantings at even distance across the fields”	B3
Skills of operators (herbicides)	“The man who sprayed the herbicide was not an expert. He did not ensure that the herbicide was sprayed evenly/completely. He left gaps and wherever the herbicide was not sprayed, the weed has remained intact. It was the fault of the labour”	B8
Skills of operator (drill)	“They did not have proper idea on how to operate the drill. When you buy the machine you get a book that has all the information like how deep to sow. But these service providers don't follow such instructions”	A1

#### 3.3.2 Constraints in water availability and access

While there were limited issues raised with accessing herbicides or spare parts for the ZT drill, irrigation was a key supplementary resource that strongly impacted implementation. There were ongoing issues in all communities with diminishing water access, especially with groundwater availability at the early establishment timing for DSR (e.g., “*The water table goes so low that the bore well goes dry. The water table falls from 10-15 m deep to 40–50 m. Around 90% of bore wells failed, so if the irrigation means have failed, how will you do this [DSR]?*” [A2]) and groundwater access (e.g., “*Many people don't have tube wells, so this is a problem, and because there is an ongoing water crisis, not everybody is willing to share their bore well water with others*” [B7]). In the context of water scarcity and increased climate variability (e.g., “*Rainfall has become the biggest problem of farmers … unpredictable weather around here in the last 4 to 5 years has ruined farmers more than anything else*” [A6]), DSR has become perceived as unsuitable (e.g., *“Last year the DSR crop failed,* *that is why nobody tried this year. It failed because it did not rain*” [B1]). However, one positive driver related to water resources is the transition to electrical pumps (e.g., “*the irrigation cost has significantly reduced after getting electricity connections. In the present scenario, it is no longer possible to irrigate our farms using a diesel pump*” [C5]).

#### 3.3.3 Access to information

In terms of information, a diverse range of information sources were identified in the communities surveyed, including newspapers; television; university staff and agricultural fairs; development project staff; personal contacts; block officers; personal fieldtrips to Punjab; visits directly from knowledgeable people; machinery companies; and internet sources (especially YouTube). However, non-DSR users appeared more likely to depend on local information and interaction through personal experiences and generally appeared less aware of DSR activities in their own communities (e.g. “*Nobody has done rice with ZT*” [C22]; “*I never went to the field. He described to me his experience that he could not get good yield from DSR*” [A4]).

The importance of projects as sources of supplementary information was also discussed by respondents. For example, projects were seen to spread information wider than business-oriented service providers (e.g. “*He [project staff] would go among farmers and encourage them, but I [as a service provider] only tell those interested people who come to me for ZT*” [A2]). Disadopters appeared much more likely to rely on projects and when removed, identified this as a driver of their disadoption (e.g. “*[Project staff] for some reason shifted elsewhere, so we stopped using these machines … [project] people weren't here, so that led to decline of self-belief … We don't know anyone else [helping farmers with DSR*]” [A5]).

### 3.4 How does communal perceptions and behaviour affect DSR use and service provision?

#### 3.4.1 Communal information systems

Issues with poor outcomes in the most recent Kharif season were prevalent in each of the communities surveyed (e.g., “*There is a farmer who did DSR in this village, but his DSR crop was a disaster. Only some of his seeds germinated and the others didn't … everybody came to know about it”* [C3]; “*Last year, we had a very bad result. In order to bring back farmer's belief in DSR, a lot of hard work has to be done*” [B2]). Beyond the poor rainfall last year leading to poor yields, addition issues were raised with: [1] a lack of technical support (e.g. “*The crux is that that first rice planting with ZT machine needs to be done under the supervision of someone who has the knowledge, otherwise no farmer will take the risk of direct sowing*” [B11]); [2] potentially unsupportive service providers (e.g. “*This [follow up with farmers] isn't my problem. A farmer comes to me to ask for direct sowing in his field. I sow his field with the machine and that is it. He pays the fee and if he needs things to be done afterwards it is his worry, not mine … My responsibility is only if seeds don't germinate in the fields. After, that it is not my responsibility*” [A6]; and [3] a lack of ability to assess reasons for failure (e.g. “*suppose a farmer has done DSR for the first time and that year he did not get good results with it because of lack of rainfall, he will never understand that it is due to poor rainfall, even as grain production was affected everywhere. Instead, he will think that transplanting through hands had been better, and now, because of machine, everything is bad"*[A6]).

The nature of social networks spreading bad news in local communities was also highlighted (e.g. “*If a farmer says to another farmer that this machine is bad, then that farmer will go to 10 more farmers and say the same thing and then those 10 farmers will go to 100 farmers and bad mouth the ZT machine. It's easier to criticize something then to say good things*” [B1]). This may be further exacerbated by negative information flow from competing service providers (e.g. “*These service providers without ZT machine do negative publicity on ZT and exaggerate the problems, and say the DSR crop failed last year and that this machine is bad*” [B1]).

#### 3.4.2 Communal belief and change mindsets

Other generic issues with community mindsets also arose, be it specific to DSR (e.g., “*They are very rigid in their beliefs and don't want to change. Transplanting rice is a tried and tested technique that they have seen and can trust. With DSR, they are still not able to trust this*” [B8]) or more generically (e.g., “*They really never think about doing anything new and are not very active. They don't like changes and also don't like to experiment*” [C7]). While use of local information providers though a lead farmer approach has been employed, this may also be overwhelming to those who are tasked with overcoming these mindsets (e.g. “*they don't understand and we don't have enough time to convince them …. You cannot make everybody understand as they are not ready to listen. Most of them are not very educated and are rigid in their beliefs and still follow what their fathers taught them*” [A6]; “*this [convincing them] involves telling the same things in different ways to each farmer which is very time consuming and I don't have enough time for all that*” [A2]). Formal extension networks were rarely mentioned as an alternative and influential information source.

#### 3.4.3 Communal physical resource management

The prevalence of small farmers and land fragmentation was an identified constraint for building a DSR customer base, due to both the logistics of mechanisation on small farms (e.g. “*In small plots, farmers can themselves do everything without the need of a ZT machine*” [A9]) and ongoing seed drop issues having a larger effect on small farmers (e.g. “*To big farmers it does not matter much if in some spots seeds fail to drop [with a ZT drill] but to small farmers these spots that remain fallow matter a lot. So small farmers with farm less than one acre usually practice traditional methods*” [A9]).

Specific to DSR service provision, issues arose with working with small holder farmers due to the size of fields (e.g. “*In small fields, it's not possible to specify the amount of seeds. In some cases, the seeds and fertilizers are less than needed and farmers have to go to shops, to buy them.* *At* *other times, farmers ask you to empty out the remaining seeds and fertilizer from the ZT machine. As a result, there are time losses … larger farmers don't do such a thing*” [B9]) or the tendency for smallholders to use lower quality inputs (e.g. *“Poor farmers opt for fertilizers that are sold at 20–25 rupees cheaper, but when the fertilizer is put into zero till machine and the weather turns misty, the fertilizer jams in the machine and only the seeds drop. The problem is that the sowing work for that day will end because of the fertilizer that gets jammed in the machine. It is difficult to remove the jammed fertilizer from the machine. It almost takes a day to get the jammed fertilizer out with a screwdriver*” [C12])

The community also seemed to identify a general movement away from rice (e.g. “*Farmers do not want to plant rice in general because of the cost of puddling, irrigation, seed preparation,* *and* *transplanting labour. Money has to be raised for these things which is not easy for the farmers. Some farmers … stopped growing rice altogether. Now they opt for wheat, potatoes and corn cultivation*” [B10]), often leading to the rental of lands (e.g. “*We do so much farming but never grow more than one acre of rice. We might give it to others, say somebody from a lower caste background, but will never do it on our own … they can do the transplanting as well as the cutting of rice crop with their hands*” [A4]). Comparative advantage of other crops like sugar cane as replacements was also discussed (e.g. “*the status is that sugarcane is the main crop while rice is in decline …. Rainfall has become the biggest problem of farmers. And the other reason is that sugarcane mill opened*” [A6]).

#### 3.4.4 Communal financial constraints

While respondents did not mention financial constraints for themselves, the financial situation within their communities was consistently mentioned as an impediment to scaling DSR, despite DSR potentially reducing costs. This related mostly to risk attitude (e.g., “*So no one can take bigger risks than farmers but like I told you earlier, the biggest problem a farmer faces is money. The farmer is unable to take the risk even after wanting to because of financial constraints*” [C19]). The differing pattern of production also potentially does not align with farmer's funding windows (e.g., “*I tell them that it’s [DSR failure] because of not spraying herbicides and watering your farm at the required time that you got such a result … They say that they didn't have the money so they couldn't do it on time*” [A3]).

#### 3.4.5 Generic service provision considerations

More so than related to DSR as a technology, constraints to service provision were likely to be non-specific to DSR highlighting that DSR may not be the primary reason for low conversion of ZT drill owners to DSR service providers (Table 5). These identified issues apply also to ZT wheat and warrant further investigation as to why these are not as constraining to other service provision activities (especially why some choose to be ZT wheat service providers but not DSR service providers).

**Table 5:** Considerations made by machinery owners before becoming a community agricultural service provider.

Constraint	Representative quotation	ID
Credit and recovery	“Farmers provide only 10% of the service fee upfront. Mostly it's on credit which will takes 1–2 years to return.… our payments keep getting delayed and on many occasions, we don't get them”	B5
Timeframes for repayment	“That is also a reason why I stopped giving agricultural services. Many farmers are not able to pay money at all for these services. If I provide my services, then that farmer will only be able to pay me once he sells off his crops. He has no other source of money to pay for my services. Only when he sells his grain will he be able to eat and pay money to the drivers. By working on credit, I lost a lot of money”	C16
Repayment culture in community	“When it came to receiving free seeds, everyone was on the line. When farmers had to payback half the amount, then half of farmers left. Now, when it's time to payback the full amount, then everyone runs away. This is the truth”	B3
Hesitance in incurring fixed costs for driver	“If I want to keep a driver, then he would charge me 400 rupees per day. Also, he would provide most of services on credit to farmers. I am busy with my own farming and then to get my payments I have to go to farmers and ask for my money”	B9
Credit affects radius of operations	“Such farmers are mostly local farmers who are mostly in the habit of not paying back. If I provide services to farmers living outside the 1 km radius, then there is no credit system”	B6
Tractor competition/oversupply	“[tractor] demand has decreased. Before, there were very few tractors but now the number of tractors have increased”	C16
Operating with small scale farmers (Transit)	“If some small farmers ask to do sowing with ZT machine in his 2 katha farm, then it does not make much economic sense for us, we will not go. The amount of money that we would get after servicing his small farm would be equal to the diesel that we used for reaching his farm”	A9
No reward for high quality services	“The farmer does not see how well the work is done by a service provider. Instead, the first thing that they care about is the cost they pay for the service”	B6
Tractor longevity	“If we use the machine for providing services, then the machine will need to be repaired in 5 years. And if I use the tractor for our own use than its life will be for around 20 years.”	C4
Reputation management (existing)	“My father has built a very good reputation and goodwill in this area, so I just want to maintain that and not ask money from people”	B8
Reputation management (Future)	“Once you get a bad name in business it's becomes very difficult”	A1
Caste Implications	“Some farmers don't want to take services from a particular person because he is from a different caste … They would instead take services from their neighbour or anybody else thinking that he will get some money because of it and that guy who is not as knowledgeable will mess up the sowing and then the farmers thinks that DSR is not good”	A2
Driver motivation	“A driver is a driver, he has no risk over his head. But if he messes it up then it will be my loss. He will just leave and move to another tractor operator”	A10
Upskilling drivers for DSR	“Once we are taught about the process, we are able to operate it without much problem. But drivers are not able to learn it quickly … I can't depend on the driver as he may destroy somebody crop and that blame will come on me”	A10

#### 3.4.6 Limited market development strategies

Despite many DSR service providers expressing a desire to help others, most did not have proactive market development strategies, mainly relying on customers seeking them out (e.g., “*I do not promote myself. If they like me, then they can call me or if not, they can call someone else*” [C11]). In fact, proactivity was potentially viewed poorly by others in the community (e.g., ‘*if I do this more [promotion], then they get suspicious that maybe I am getting profit from this … Take the example of your family, if you keep pushing someone to do something. Then they start wondering, why is he putting so much pressure on me?*"[B7]).

### 3.5 How do government programs affect the enabling environment for DSR?

Nearly unanimously, respondents had negative assessments of government programs and their implementation (e.g., “*The government policies also don't help the farmers. The government passes policies and laws on paper, but the ground reality is completely different … Everything that comes from Central Government disappears by the time it reaches Bihar*” [C19]). This came from a broad range of issues that are summarised in Table 6.

**Table 6:** Constraints perceived in government program implementation identified by respondents (note these are perceptions and not confirmed constraints).

Issue	Representative quotation	ID
Not perceived as government promoted	“Government has so far failed to conduct any demonstrations for DSR”	B3
Unfamiliarity with government processes	“If I tell them that the block (extension service) is giving kits to farmers, then they won't take it as they can't understand things … they don't understand the rules and regulations of the government”	B5
Motivated advisors	“We have government officials, who are posted as advisor to farmers. These people are focused more on their government jobs and hardly interact and tell farmers about anything. Government doesn't care much about the farmers, and their problems and prosperity”	A7
extension for financial not informational purposes	“Farmers get rice seeds [for DSR] from the block but these officers just tell them that you will get back your cost of seeds as the amount will be transferred to your account but they do not teach or train them on how to use it”	A3
Timing issues	“It [government] cannot provide the correct things at the times needed. Only after I sowed moong dal [seeds were provided] and similarly Dhaincha (*Sesbania* sp) seeds came after Dhaincha was cut down in fields and rice seeds came after seeds sowing seeds in seed beds”	B3
Corruption (electrification)	“electrification of farms is taking place. But even for that we have to pay bribes. For a new connection we have to pay 2000 rupees bribe and for getting the meter another 500 rupees bribe”	B6
Corruption (subsides)	“In order to get subsidy finalized, they have to pay little bit commission to officials. Only, after, that subsidy will be given back”	B9
Middlemen	“We think that we will get it but after delay, we end up getting nothing. And then there are middlemen who try to swindle money out of us and want commission … The government might be giving but we don't end up getting anything”	C11
Siphoning	“The main thing is that government doesn't have agenda for farmer's development and wellbeing. If there's anything, those good plan and policies remains stuck in the pocket of government officials”	A7
Quality and Trust	“They distributed some seeds in the village but those seeds were old due to which it did not germinate. The government should think about the state of farmers before distributing old seeds to them”	C16
Functionality and practicality of financial support	“They said that fertilizers will be provided if farmers deposit 10,000 into their account and they will get 11,000 rupees in return. A farmer doesn't have 10,000 to deposit into the bank so that he can get fertilizer and 1000 rupees … and the subsidy will be provided only after 3 months. By then the entire farm will dry up just to earn extra 1000 rupees”	C16

More specifically, some programs were seen as inefficiently implemented or with adverse outcomes. For instance, the MNEREGA (Mahatma Gandhi National Rural Employment Guarantee Act) was seen to be driving up labour costs (e.g., “*there are labour problems. If government can stop the loot that is happening in MNEREGA and move labour from there to farmers, then something could happen*” [B3]). The Bihar rural electrification scheme was both strongly discussed and linked back to DSR by respondents (e.g., “*It is because of lack of irrigation that we are not able to do DSR. Bihar government's goal is to connect each farm with electricity. But so far nothing much has happened in our village regarding this initiative*” [A1]). For those that have been connected, the erratic nature of electricity provision was a common issue (e.g., “*Even after we obtained power supply, it is very erratic and of late it is getting even worse and we are not able to irrigate. If the power is there for an hour, then next 2 hours* *it goes away, then it comes back for an hour and goes away for next 3 hours*” [B6]). There was also some acknowledgement that electrification may not be a ‘silver bullet’ for DSR (e.g., “*the level of bore well water is limited, even with help of electricity*” [A8]).

In terms of DSR specific government programs, there was a lack of common understanding on if DSR subsidy programs were still active with some farmers receiving payments while others not (e.g., “*From three years people from the block also enquired with us about those farmers who took my ZT machine for sowing. But, as its not part of government program, slowly they stop taking interest in my machine too*” [A7]). Functionality was often questioned (e.g., “*Nobody gets any money. The government officials never give out any money to any service providers. If a service provider says he is getting some incentive for providing ZT or DSR service, he is lying and being dishonest. We should be getting money but the truth is nobody gets any money. It's been like this since the last 3–4 years*.” [A2]). The machinery subsidy was also perceived as a hassle of procedures (e.g., “*farmers don't want to go through the entire process of registering. And all the other formalities to get the subsidy*” [B5]; “*I am sure there is a subsidy if we need to buy, but I was in a hurry, when I bought it. I did not want to get tangled in all the formalities required for a subsidy*” [C1]).

## 4 Discussion

**Fig. 3:**
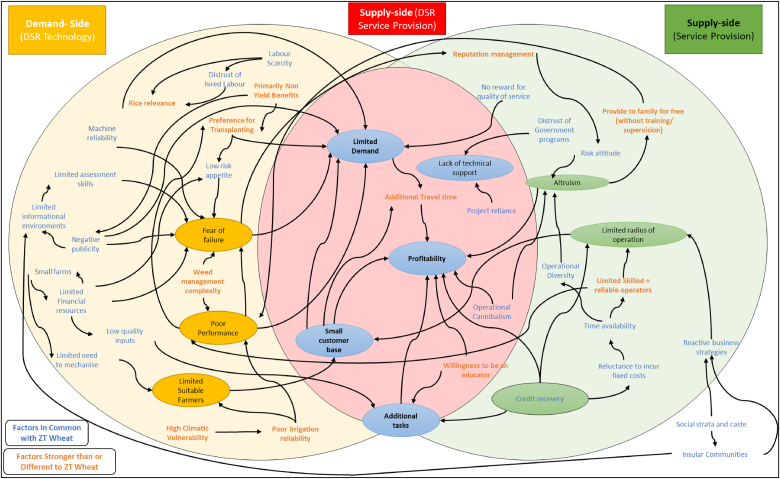
Three interrelated questions identified for exploration to diagnose DSR adoption constraints.

Our results highlight a complex web of constraints driving the limited utilisation of ZT drills by owners for rice in the Kharif/monsoon season. These are primarily based around two interrelated yet independent decision process that highlight the need to ask the correct set of questions when exploring agricultural service provision: [1] On the demand side, is there a problem with the technology (DSR)? and [2] On the supply-side, is there a problem with agricultural service provision. The subsequent interaction of these two questions then relates to the third question: [3] On the supply side, is there a problem with providing services with the technology (DSR; Fig. 3). The thought processes respondents identified are summarised across these three questions in Fig. 3. In particular, results highlight that the technology should not be the only or even primary consideration when considering service provision decision making.

### 4.1 Is the problem with the technology (DSR)?

#### 4.1.1 Perceived benefits of DSR (and a lack of yield benefit)

Respondents highlighted a board recognition of the benefits of DSR (from lowering production costs, reducing water use, reduced labour dependency, and complementary benefits with the following wheat crop), yet mostly did not identify a positive change in yield. Many identified slightly lower yields, but that this was compensated by cost savings. This is mostly consistent with the literature that highlights DSR yields remain mostly stable compared to PTR when well managed and when critical inputs such as irrigation and herbicides are guaranteed. Without these conditions there are substantial risks to yield penalty (Pandey and Velasco, 2002; Farooq et al., 2011; Xu et al., 2019; Singh et al., 2020). Likewise, it confirms that labour and water constraints and savings in production cost are stronger drivers of DSR uptake than changes in yield. Importantly, this is distinctfrom ZT wheat which does show substantial yield advantages compared to conventional production systems (Keil et al., 2020), though this yield increase may be confounded by the effects of early wheat sowing enabled through ZT (and not ZT itself as a practice). None the less, this indicates a need for different extension and marketing strategies to be employed to highlight why farmers should consider a transition to DSR and importantly, what requirements are integral to successful outcomes (including irrigation, land type, weed management and herbicide application skills in lowland agro ecology).

#### 4.1.2 Irrigation access and requirements (and erratic rainfall)

While respondents positive to DSR did identify overall benefits, those who tended to be more negative highlighted concerns about poor performances of DSR in their communities, which had relationships with the complexities of DSR as compared to PTR (e.g., weed management and irrigation requirements). This was further compounded by recent seasonal experiences of erratic rainfall of which the effects are felt more due to unreliable irrigation facilities, again more strongly influential than in non – puddled wheat systems. Given limited tube well access and erratic rainfall (Najmuddin et al., 2018) and a decreasing water table (Jain et al., 2021) are common throughout Bihar, these results indicate that DSR in Bihar is likely to be widely constrained by this irrigation constraint. While the Government of Bihar rural electrification schemes is attempting to address this, these remains an overall distrust of the scheme that is perceived to have issues of reliability, confirming the reliability concerns found in Oda and Tsujita (2014).

#### 4.1.3 Distrust of hired labour and skilled operators

Poor performance of DSR was also linked a generic distrust of hired labourers, as well as a specific identification of a lack of skilled DSR operators and corresponding seed drop issues with the ZT drill, a relatively well documented issue across ZT drill use regardless of crop (Groot et al., 2019; Darbas et al., 2020). While a distrust of hired labour for transplanting was present, the distrust of drill performance was usually a stronger consideration, meaning resolving ‘gapping’ of seed drop could turn this constraint into a driver of DSR uptake. This seems to be more important in rice systems as opposed to wheat, likely due to: [1] the wheat seeding rate being comparatively high; [2] that wheat seed is less sensitive to seeding depth; [3] that the winter season has less risk of erratic rainfall and implications of water stress or excess water due to excessive rainfall; and [4] that transplanting rice has a high establishment rate compared to DSR.

Service providers also identified that bad publicity also influenced community perception on DSR, related to the adage that ‘bad news travels faster than good news’ and that some non ZT service providers were spreading disinformation to further engrain the demerits of DSR. These issues were key to feeding a communal narrative of DSR as unproven and likely to lead to crop failure, often framed as total crop failure. Given these information flows place services providers against service providers in a competitive business environment and that disinformation about DSR is prevalent within communities, DSR service providers are likely to find it hard to break this disinformation chain. Hence, it may require concerted efforts from ‘trusted outsiders’ such as projects and government sources to overcome these issues (and these efforts should focus more on information than input provision).

#### 4.1.4 Weed management issues

Weed management concerns are a well-known DSR adoption deterrent (Kumar et al., 2013; Rao et al., 2017), with respondents perceiving a substantial transition required from a relatively simple ‘tillage and puddling’ regime to a more complex herbicide-based management system. Respondents highlighted additional complexity in selection, application, weather forecasting, use of specific equipment, mixing and storage techniques and other relatively new and knowledge intensive requirements, that were often made more complex in the monsoon season as compared to the winter season. The complexity of herbicide selection and use was also identified as a key constraint by Kapil Singla et al., (2012), and aligns with the assertion of DSR as a more complex and knowledge intensive system by Singh et al., (2010). This knowledge gap highlights that additional resources will need to target capacity development of potential adopters, whereby they understand DSR not as a change in planting system but a transition of production system which requires additional management skills. Additional research should focus on how strategic minimum tillage systems may be able to overcome some weed management issues as compared to complete ZT systems. These results also highlight the need to train not just service providers, but farmers and local labourers on weed management in DSR alongside best bet herbicide application techniques and the safe handling of herbicides to achieving higher weed control efficiencies.

#### 4.1.5 Lessening relevance of rice

Some respondents asserted that rice may be losing relevance as the dominant preference for the Kharif/monsoon season, driven by irrigation, labour scarcity and labour reliability. This provides further support for other studies suggesting a reduction in rice prevalence in India and globally (Mishra et al., 2017), driven by crop diversification, globalisation, and rising incomes (Pingali et al., 2019).

### 4.2 Is the problem with agricultural service provision?

Overwhelmingly, the main constraints raised by respondents were not related to DSR but were related generically to agricultural service provision, and this was the main driver in a hesitance to provide DSR (or any) services in their community.

#### 4.2.1 Provision of services on credit

Nearly all respondents mentioned issues with providing services to community members on credit, and the time spent in trying to collect dues owing. This trend has also been reported by agricultural service providers in Punjab (Singh, 2017). This issue can be traced back to a constraint with working resource poor smallholder farmers with considerable limitations in their financial constraints. Given that smallholders in Bihar are known to have limited collateral and limited formal credit access (Najmuddin et al., 2018), the burden of credit default is effectively passed from the formal credit sector to informal credit (i.e., service providers themselves). Respondents indicated that this was a key driver in the decision not to provide agricultural services, as well as limit the radius of their service provision activities if they did choose to provide services. This issue may require innovative approaches to subsidy schemes that directly pay service providers rather than farmers for DSR planting services, to ensure that DSR can be accessed by a wider spectrum of socioeconomic groups.

#### 4.2.2 Passive market development strategies

None of the respondents in this study identified a proactive promotional strategy for their agricultural service provision business, with most expecting that customers would come to them directly. This was in part a reflection of cultural values in the region where marketing may lead to negative communal perceptions. This trend was also evident in Punjab, where service providers did not promote their agricultural services beyond personal contacts (Singh, 2017). Given DSR will require service providers to educate and motivate their customer base, this appears potentially problematic to the wider scaling of DSR. These results suggest the importance of demand creation for DSR to be led by ‘trusted outsiders’ to support service provision. Respondents identified that outsiders such as projects were integral to scaling of DSR, because they have the ability to promote and teach within the community more so than individual service providers.

#### 4.2.3 Time constraints and altruism

Time constraints were primarily related to a livelihood prioritization decision between profitability and altruism. In most cases, time was limited which reflected that most tractor owners have their own sizable farms to manage. This led to limited time to provide subsequent services to others. Hiring other operators was also problematic due to a lack of skilled operators (Singh, 2017; Groot et al., 2019) and limited trust in them to perform highly which may lead to reputational damage. Service providers in this study tended to have altruistic motivations that meant they were willing to sacrifice some level of profitability to help others in their community. This trend was also highlighted by Keil et al. (2016) who found that ZT wheat service providers tended to not be larger, but medium sized farmers who were large enough to own a tractor, but still have labour resources available to make use of service provision opportunities. This may confirm that subsidies to encourage service provision could be targeted to such groups.

### 4.3 Is the problem with DSR service provision?

#### 4.3.1 Difficulties in farmer change management

The additional complexities of DSR appear to compound issues with farmers managing and evaluating production options. Respondents indicated that their potential customer base were perceived to have limited education, limited sources of information and a lack of critical reflection on why DSR may fail (e.g. that erratic rainfall or poorly skilled operators, not DSR itself may be the cause of recent poor performance). Our result indicate that information systems remain insular and limited, which appears to further constrain positive DSR evaluations. Such findings are consistent with Dessart et al. (2019) who highlight risk-aversion in farming populations is strongly related to accessible and reliable information, as well as Chavas & Nauges (2020) who explain how the complexities of learning about innovations which are dependant on site specific variables (like DSR) slow adoption processes. Given that DSR is more knowledge intensive and complex within the context of insular information systems, further efforts will again need to focus on ‘trusted outsiders’ improving informational systems that not only highlight the benefits of DSR (e.g. demonstrations) but focus on how to select appropriate fields for DSR and apply new techniques to ensure successful DSR implementation (i.e. more intensive field schools, ongoing trainings and interactions).

#### 4.3.2 DSR requires additional and often unfunded tasks

The primary issue with DSR service provision was related to the need for additional tasks as compared to other agricultural services. These constraints were traced to issues working with smaller, less resourced smallholder farmers. For instance, under resourced farmer were likely to use lower quality inputs that were not compatible with the ZT drill or due to small field size would require small volumes of seed and fertiliser returned from the drill after planting. There were also issues with providing services with the ZT drill on small-sized fields and the transit time involved in servicing dispersed fields throughout a community, more apparent with the smaller customer bases that exist for DSR than ZT wheat. Most respondents also identified a limited DSR customer base, whereby farmers will need assured irrigation and an open mindset which were both in short supply. This meant that an already small pool of potential customers was further limited, and overall demand for services was constrained. While small land holdings and travel times are a common issue that lead to issues with financial viability (Singh, 2017; Yadvinder-Singh et al., 2020), such issues are likely to be further increased with DSR considering further limited pools of customers and already constrained radii of operation. Marketing DSR as part of a combined service package (e.g. including herbicide spraying and follow up services for first 50 days) could enable pricing for such currently unfunded services.

#### 4.3.3 Business risk and reputation

Providing DSR services in the context of widespread community perception of poor performance and variable water resource access was perceived by respondents to put their businesses at risk of reputational damage. Given that DSR is a small subset of the overall business portfolio for the majority of service providers, the risk to reward ratio was perceived as limited. Given the context of expanding competition for customers and the need for continual customer satisfaction to ensure economic viability (Bitzer et al., 2016), risking poor DSR outcomes on recipients’ lands could have large economic ramification for their whole business models and was hence avoided unless altruism was a motivation for providing DSR services. These issues were especially compounded due to the successful outcomes in DSR fields being related not only to their planting service quality, but the numerous other tasks outside of their current business models that are reliant on farmer skills and resources (e.g. irrigation, rainfall and herbicide use). The effectiness of these activities will ultimately determine service recipient and community DSR evaluations. Like with overcoming additional unfunded tasks, a packaging of follow up services may be useful in ensuring successful DSR outcomes and more positive communal evaluations.

#### 4.3.4 DSR/ZT and competing business opportunities and investments

A key issue raised by respondents related to the financial implications of reduced tillage passes that in turn decreased income, especially when machinery such as the rotovator has already been purchased specifically for business income. Given economic business models rely on tillage passes and a reduction in passes is one promoted benefit of DSR, internal cannibalism of economic activity is a logical perception. With increasing competition for agricultural services and limited customer bases, DSR may not be the most economically productive activity to provide. This confirms similar finding on the need to offset service provision of ZT wheat in Haryana with other tractor service provision activities (Erenstein et al., 2007) as well as the comparative probability of other machinery such as the rotovator (Hassan et al., 2017). To achieve profitability, a higher volume of customers may be needed in which the current customer base is constrained. Hence, packaging of various services and machinery may help to resolve this constraint.

#### 4.3.5 Altruism mindset

While resources available to provide services were important, more so a mindset of altruism was key to a willingness to provide DSR services. This meant that DSR service providers needed to prioritise community benefits over their own profitability, due to additional tasks, reputational risk and competing business opportunities. With the limited time they had, they needed to be willing to use that time not for their personal good but for communal good. This learning is important in future interventions – for instance ensuring that targeting of interventions includes consideration of available time and altruistic mindsets for inclusion in subsidy programs. Over the long term, there may also be value in promoting the positive aspects of servicing your community to build more positivity towards agricultural service provision as a communal good that might increase a service providers status as a community leader.

## 5 Conclusions

While DSR has numerous potential benefits to South Asian smallholder farmers, it's out scaling has been limited by both a lack of demand by farmers and limited supply of DSR services by drill owners, in contrast to ZT wheat which has been by comparison rapidly expanding. In exploring the thought processes of ZT drill owners, it becomes apparent that complexities exist for respondents in both implementing DSR and providing DSR services within their communities. It would appear from those thought processes that the transition from PTR to DSR is more complex than transitioning from conventional tillage to ZT wheat systems. At the core of this change process is that puddling is a proven and trusted method of production and that DSR has not been able to displace this firm belief that tillage and puddling systems are reliable and resilient in the given production context. This in part reflects ongoing erratic rainfall and irrigation constraints, as well as increased complexity in herbicide and weed management in DSR. Given that no yield benefit is evident in transitioning to a more complex system, transitioning farmers are likely to need a more intensive promotional program focused on the various non-yield benefits of DSR. This is in contrast to ZT wheat where yield benefits are experienced alongside cost savings as driving factors in substantial long term promotional programs and farmer uptake. Given the more complex promotional context, respondents also indicate that service providers themselves are not willing (or often culturally able) to be DSR promoters, due to prevalent socio-cultural constraints and potential reputational risk involved in promoting DSR to potential customers. Instead, ‘trusted outsiders’ such as project are needed to increase awareness and understanding of DSR system benefits and management requirements.

In terms of targeting potential service providers, interventions should consider targeting those with altruistic motivations and available time (i.e. medium sized farmers), ensuring they are able to action DSR given the additional unfunded tasks that are involved in DSR service provision. Such service providers should also be trained on DSR agronomy and to identify appropriate fields and agro-ecological contexts to increase the beneficial outcomes of suitable fields whilst avoiding fields that are likely to have production constraints or farmers who may be unwilling to adapt to changed DSR management protocols. This acknowledges that DSR will not be widely suitable to all farmers under current circumstances in rainfed system in Bihar. Enacting this will also reduce the potential reputational risk involved in providing DSR services, particularly if promotional efforts are led by outside influences such as projects who are seen to be technically supporting service providers in the initial years. This will require generic business management skills to be integrated into service provider training programs.

Given the increased risk involved in farmers adopting DSR, two alternative pathways may be considered for service providers and the communities they exist in. Firstly, to de-risk DSR, cooperatives and clustering approaches could be employed in order to engage suitable and sizable areas for DSR implementation. In this way, risk is shared by cooperative or cluster members and greater control is enabled for irrigation services. This also creates demand aggregation to overcome current issues with servicing dispersed customers. Additionally, services providers should be encouraged to market a packaging or services beyond planting (including irrigation, spraying and nutrient management operations) as part of a ‘single-kit’ product. Assuming that service providers are up-skilled sufficiently to provide these service and financially able to afford the additional implements, this will form part of an additional de-risking mechanism and enable some of the currently unfunded DSR tasks to become a costed activity within their business.

Overall, this research identifies a series of thought processes applied by machinery owners which are analysed within a novel qualitative framework that disentangle the reasoning behind underutilisation of their purchased ZT drills in the monsoon season. This highlights the additional complexities of DSR, of which some potential solutions are identified, and in particular that it should not be assumed that DSR will scale in the same way as ZT wheat has. The value of exploring generic service provision constraints alongside technology also provides added value to exploring decision making, highlighting that many of the constraints experienced are not technology related. While this research frames DSR service provision as a case study, the analysis framework proposed also provides a potentially useful tool to further exploration of farmer and service provision decision making and resource allocation more broadly.

## Credit author statement

**Brendan Brown:** Conceptualization, Methodology, Formal analysis, Data curation, Writing – original draft, Writing – review & editing, Visualization **Arindam Samaddar:** Conceptualization, Methodology, Formal analysis, Writing – original draft, Writing – review & editing **Kamaljeet Singh:** Investigation, Data curation **Ava Leipzig:** Writing – original draft, Writing – review & editing Anurag Kumar: Investigation **Pankaj Kumar:** Investigation **Deepak Kumar Singh:** Investigation Ram Malik: Writing – review & editing **Peter Craufurd:** Writing – review & editing, Supervision **Virender Kumar:** Conceptualization, Writing – review & editing, Supervision **Andrew McDonald:** Conceptualization, Writing – review & editing, Supervision, Funding acquisition.

## References

[bib1] Anibaldi R. (2021). Theoretical underpinnings in research investigating barriers for implementing environmentally sustainable farming practices: insights from a systematic literature review. Land.

[bib2] Bhardwaj S., Sidana B.K. (2017). Factors influencing adoption of direct seeding of rice technology in Punjab agriculture. Int. J. Innovative Res. Sci.Technol..

[bib3] Bitzer V. (2016).

[bib4] Brown B., Nuberg I., Llewellyn R. (2017). Negative evaluation of conservation agriculture: perspectives from African smallholder farmers. Int. J. Agric. Sustain..

[bib5] Brown B., Nuberg I., Llewellyn R. (2017). Stepwise frameworks for understanding the utilisation of conservation agriculture in Africa. Agric. Syst..

[bib6] Brown B., Paudel G.P., Krupnik T.J. (2021). Visualising adoption processes through a stepwise framework: a case study of mechanisation on the Nepal Terai. Agric. Syst..

[bib7] Chavas J.P., Nauges C. (2020). Uncertainty, learning, and technology adoption in agriculture. Appl. Econ. Perspect. Pol..

[bib8] Darbas T. (2020).

[bib9] Dessart F.J., Barreiro-Hurlé J., Van Bavel R. (2019). Behavioural factors affecting the adoption of sustainable farming practices: a policy-oriented review. Eur. Rev. Agric. Econ..

[bib10] Devkota R. (2020). “Responsible agricultural mechanization innovation for the sustainable development of Nepal's hillside farming system. Sustainability.

[bib11] Erenstein O., Farooq U. (2009).

[bib12] Erenstein O., Malik R.K., Singh S. (2007).

[bib13] Farooq M. (2011). Rice direct seeding: experiences, challenges and opportunities. Soil Tillage Res..

[bib14] Groot A.E. (2019). Business models of SMEs as a mechanism for scaling climate smart technologies: the case of Punjab, India. J. Clean. Prod..

[bib15] Guru P.K. (2018). Mechanical transplanting of rice in India: status, technological gaps and future thrust. ORYZA Int. J. Rice.

[bib16] Hassan A. (2017). Role of agricultural services providers (ASPs) in enhancing the productivity of crops in district faisalabad article. Int. J. Adv. Multidisciplinary Res..

[bib17] Jain M. (2021). Groundwater depletion will reduce cropping intensity in India. Sci. Adv..

[bib18] Kakumanu K.R. (2019). Adaptation to climate change and variability: a case of direct seeded rice in Andhra Pradesh, India. J. Water Climate Change.

[bib19] Kapil Singla B.R. (2012).

[bib20] Kaur J., Singh A. (2017). Direct seeded rice: prospects, problems/constraints and researchable issues in India. Curr. Agric. Res. J..

[bib21] Keil A. (2020). Zero-tillage wheat provides stable yield and economic benefits under diverse growing season climates in the Eastern Indo-Gangetic Plains. Int. J. Agric. Sustain..

[bib22] Keil A., D'Souza A., McDonald A. (2016). Growing the service economy for sustainable wheat intensification in the Eastern Indo-Gangetic Plains: lessons from custom hiring services for zero-tillage. Food Security.

[bib23] Keil A., Mitra A. (2017).

[bib24] Kumar A. (2015). Productivity and economics of direct seeded rice (Oryza sativa L.). J. Appl. Nat. Sci..

[bib25] Kumar V. (2013). Weed management strategies to reduce herbicide use in zero-till rice–wheat cropping systems of the indo-Gangetic Plains. Weed Technology. 2017/01/20.

[bib26] Kumar V., Ladha J.K. (2011). Direct seeding of rice. Recent developments and future research needs. Adv. Agron..

[bib27] Maharjan A., Bauer S., Knerr B. (2012). Do rural women who stay behind benefit from male out-migration? a case study in the hills of Nepal. Gend. Technol. Dev..

[bib28] Malik R.K., Kumar V., Yadav A. (2015).

[bib29] Mishra A.K., Khanal A.R., Pede V. (2016). Agricultural and Applied Economics Association’s 2017 AAEA Annual Meeting, Chicago, IL, July 30-August 2, 2016.

[bib30] Najmuddin O. (2018). Low water productivity for rice in Bihar, India-A critical analysis. Water (Switzerland).

[bib31] Oda H., Tsujita Y. (2014).

[bib32] Pandey S., Velasco L. (2002).

[bib33] Pingali P. (2019).

[bib34] Rao A.N., Sparks D.L. (2017). Chapter Two - Preventive Weed Management in Direct-Seeded Rice: Targeting the Weed Seedbank,.

[bib35] Saharawat Y.S. (2010). Evaluation of alternative tillage and crop establishment methods in a rice-wheat rotation in North Western IGP. Field Crop. Res..

[bib36] Singh M. (2020). Intercomparison of crop establishment methods for improving yield and profitability in the rice-wheat system of Eastern India. Field Crop. Res..

[bib37] Singh S. (2017). How inclusive and effective are farm machinery rental services in India? Case studies from Punjab. Indian J. Agric. Econ..

[bib38] Singh S.P., Paikra K.K., Aditya S. (2019). Direct seeded rice: prospects, constraints, opportunities and strategies for aerobic rice (Oryza sativa L) in Chhattisgarh - a review. Int. J. Curr. Microbiol. Appl. Sci..

[bib39] Singh V.P. (2010).

[bib40] Targeting T. (2014).

[bib41] Xu L. (2019).

[bib42] Yadvinder-Singh (2020).

